# Exploring the Feasibility of Cell-Free Synthesis as a Platform for Polyhydroxyalkanoate (PHA) Production: Opportunities and Challenges

**DOI:** 10.3390/polym15102333

**Published:** 2023-05-17

**Authors:** Huaming Dong, Xue Yang, Jingjing Shi, Chunqiao Xiao, Yanfei Zhang

**Affiliations:** 1 School of Environmental Ecology and Biological Engineering, Wuhan Institute of Technology, Wuhan 430205, China; donghm@tib.cas.cn; 2 Tianjin Institute of Industrial Biotechnology, Chinese Academy of Sciences, Tianjin 300308, China; yang_x@tib.cas.cn (X.Y.); shijj@tib.cas.cn (J.S.); 3 National Center of Technology Innovation for Synthetic Biology, Tianjin 300308, China

**Keywords:** cell-free synthesis system, Polyhydroxyalkanoate (PHA), PHA synthesis pathway, multienzyme cascade system

## Abstract

The extensive utilization of traditional petroleum-based plastics has resulted in significant damage to the natural environment and ecological systems, highlighting the urgent need for sustainable alternatives. Polyhydroxyalkanoates (PHAs) have emerged as promising bioplastics that can compete with petroleum-based plastics. However, their production technology currently faces several challenges, primarily focused on high costs. Cell-free biotechnologies have shown significant potential for PHA production; however, despite recent progress, several challenges still need to be overcome. In this review, we focus on the status of cell-free PHA synthesis and compare it with microbial cell-based PHA synthesis in terms of advantages and drawbacks. Finally, we present prospects for the development of cell-free PHA synthesis.

## 1. Introduction

Traditional petroleum-based plastics possess excellent material properties, including lightweight, stability, durability, economic feasibility, and other desirable material characteristics. These features enable them to be synthesized and processed into various strength materials and molded into different shapes. These properties have made them an attractive option for utilization in construction, packaging materials, computer equipment, automotive components, medical devices, and other fields [1,2,3,4,5,6,7]. However, the extensive use of petroleum-based plastics has resulted in serious pollution to land and sea over the past few decades as large-scale production and inappropriate waste management strategies have inevitably negatively impacted the environment [8]. Thus, there is an urgent need for wise alternatives to using petroleum derivatives to reduce dependency on finite fossil resources while limiting pollution and CO_2_ emissions [9].

One attractive solution to this issue is scaling up the production and application of microbial-synthesized bioplastic polyhydroxyalkanoates (PHAs). PHAs are biobased materials that can be produced by approximately 40% of prokaryotic strains that are capable of accumulating PHA biopolymers [10,11]. The bacterial stains primarily responsible for PHA production, including *Ralstonia eutropha* [12] and *Pseudomonas* sp. [13], have demonstrated the ability to synthesize PHAs during the nongrowth stage with minimal nutrient requirements [14]. However, it is worth noting that natural PHA producers typically exhibit lower yields [15]. Over the past decades, developments in industrial biotechnology and metabolic engineering have demonstrated tremendous potential in exploiting different PHA biosynthetic host strains via natural or heterologous metabolic pathways to enhance intracellular PHA content. While the numbers of successful cases continues to grow [13,16], only a small proportion of the resulting products are economically viable for large-scale production. As such, considerable effort is still required to bring them to market.

The complexity of the metabolic system in the cell factory poses the greatest challenge to PHA synthesis. It is very hard to balance the intracellular flux in the best way to satisfy the target synthetic pathway while sustaining the host’s needs for growth and reproduction [17]. This leads to a series of challenges, including low yield and conversion, high costs of raw materials and energy, and poor material properties. The crucial problem lies in the fact that the current state-of-the-art technology is unable to cater to the demands of both mass production and tailored material properties. Additionally, the separation or postprocessing of PHA products is constrained by the properties of the PHA product itself and the bacterial cell wall. The scaling up of PHA synthesis from laboratory to industrial levels requires careful consideration of carbon source cost issues and the feasibility of fermentation conditions [18]. The intracellular synthesis of PHA with cofactors (e.g., ATP, NAD^+^, and CoA) requires a substantial amount of energy, leading to an imbalance in resource allocation (e.g., electron, CoA, and ATP fluxes) and limiting the feasibility of achieving high PHA yields with the complete synthesis of the target product. Consequently, low volumetric productivity may be observed. In addition, the intricate nature of cellular regulation can impede the implementation of high-yield pathways designed for intracellular PHA synthesis, making it difficult to assign metabolic flux according to the design [19,20].

To address the challenges faced in microbial cell-based polyhydroxyalkanoate (PHA) synthesis, several strategies have been vigorously developed. These include the development of newly engineered strains [21,22], the utilization of inexpensive substrates [14], and improved extraction methods [23]. Despite these efforts, the high-cost bottleneck in PHA synthesis has not yet been substantially overcome. As an alternative approach, cell-free systems have rapidly emerged in the synthesis of PHAs and other products. By disconnecting PHA synthesis from the generation of bacterial biomass, in vitro systems can overcome the challenges associated with microbial cell-based PHA synthesis. Through the reconstruction of the PHA synthesis pathway using cell lysates or purified enzymes, in vitro systems allow for precise detection of metabolite concentration, regulation of enzyme activity, and the control of flux distribution [24,25]. In recent years, cell-free synthesis has also emerged in combination with energetic and cofactor regeneration techniques such as electric or light energy-based methods. In addition, a range of non-natural pathways for PHA and its precursors have been shown to be compatible with in vitro systems. In this review, we focus on the current status of in vitro PHA synthesis, comparing its advantages and drawbacks to microbial cell-based PHA synthesis, and offer perspectives and outlooks on cell-free PHA synthesis.

## 2. Overview of PHAs

PHAs are a series of structurally diverse storage polyesters that are accumulated by various bacterial species and stored intracellularly in the form of granules [26]. They primarily act as carbon and energy storage compounds to sustain cell survival during starvation [27]. In 1926, Lemoigne [28] first discovered these biological polymers. Since then, PHAs have attracted significant commercial and research interest in the green polymer market due to their desirable properties such as biocompatibility, biodegradability, and diversified chemical structures. PHAs have been recognized as a green alternative to conventional petroleum-derived plastics [29].

Based on the number of carbon atoms present in the *(R)*-HA monomer unit, PHA polymers can be broadly classified into three types: short-chain-length PHAs (scl-PHA, C3-C5), medium-chain-length PHAs (mcl-PHA, C6-C14) and long-chain-length PHAs (lcl-PHA, containing more than 14 carbon atoms) [30]. Many bacteria can produce scl-PHA, including the model species *Cupriavidus necator* (also known as *Ralstonia eutropha*) [31], while mcl-PHA producers are mainly found in *Pseudomonas* sp. [32,33,34]. The monomer composition of PHA polymers defines most of the physical–chemical properties of the material, which is a determining factor in its engineering application, for example, mcl-PHA and its copolymers are suitable for a range of biomedical applications requiring flexible biological materials [32]. Additionally, PHA can be synthesized in different forms depending on the bacterial species and the substrate provided. These include homopolymers that are composed of only one type of monomer [35], random copolymers [36] that contain two or more different monomers, and block copolymers [37] that are made up of at least two homopolymers connected by covalent bonds.

## 3. Current Challenges in Synthesizing PHAs In Vivo

Despite significant progress having been made in the development of recombinant PHA-producing strains [38], obtaining PHA in large quantities at low cost remains a challenge. This is primarily due to the high cost of the carbon source used for PHA production through microbial metabolism as well as the energy-intensive separation and extraction process [39].

### 3.1. Reducing High Costs Is Difficult

The cost of synthesizing PHA through microbial fermentation remains a significant challenge, primarily due to the high energy consumption and maintenance costs associated with the production process. The commercial production of PHA is usually achieved through sterilized fermentation, which requires the use of sterile equipment and media to prevent the growth of unwanted microorganisms, resulting in a production cost that is five to ten times higher than that of traditional plastics [40,41]. Moreover, the laboratory-scale synthesis of PHA usually requires the addition of antibiotics in the culture to prevent bacterial contamination, which is impractical when scaling up to industrial production. To address this challenge, researchers have explored thermophilic or halophilic fermentation processes. The thermo-tolerant fermentation process operates at 50–60 °C, reducing sterilization costs, and allowing efficient semi-continuous and continuous modes while generating metabolic heat that can disperse to heat the fermentation tank, resulting in energy savings. Additionally, thermo-tolerant fermentation reduces sterilization costs and can operate efficiently in semi-continuous and continuous modes due to the high temperature, which most microorganisms cannot survive [42]. Similarly, halophilic bacteria can grow in high salinity waste carbon sources such as whey permeate, which contains about 200 g/L sodium chloride, and reduces the risk of microbial contamination, allowing for efficient PHA synthesis and release [43,44]. However, it should be noted that specialized fermentation equipment is required for these processes, leading to increased equipment and maintenance costs [45].

**Figure 1:**
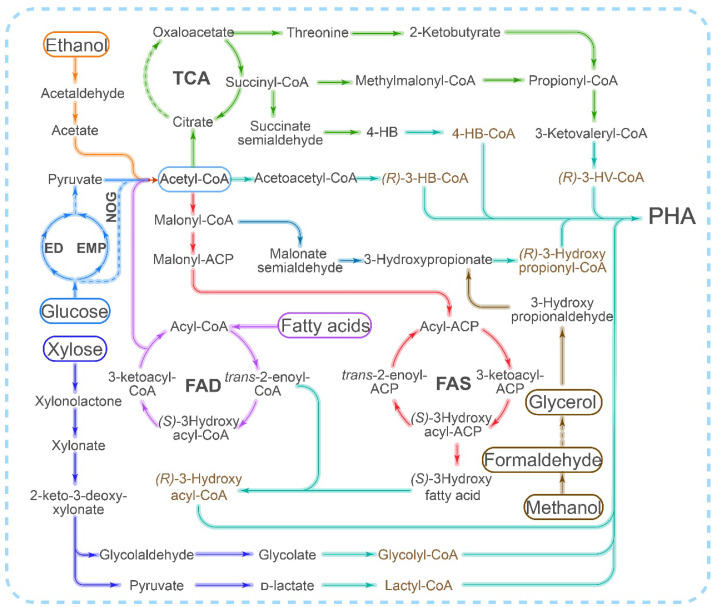
Different pathways for PHA synthesis utilized by different carbon sources. ED: Entner–Doudoroff pathway; EMP: Embden–Meyerhof–Parnas Pathway (glycolytic pathway); FAD: fatty acid degradation pathway (beta-oxidation pathway); FAS: fatty acid synthesis pathway; TCA: tricarboxylic acid cycle; NOG: nonoxidative glycolysis pathway.

Another limiting factor for the microbial fermentation synthesis of PHA is the availability and high cost of carbon sources. According to estimates, the cost of carbon sources, such as glucose, accounts for 30–40% of the total production cost [46]. Therefore, recent studies have focused on utilizing low-cost carbon sources for PHA synthesis, such as waste oil, fiber, and one-carbon resources, to reduce production costs. Additionally, researchers have been exploring ways to reduce carbon loss in glucose metabolism, such as exploring alternative pathways, including the NOG pathway and the threonine bypass [47] (Figure 1). Furthermore, some studies have investigated the use of alternative carbon sources for PHA production, such as ethanol, glycerol [38,48], and xylose [49] (Figure 1).

The third challenge in synthesizing PHA is the high cost associated with downstream recovery processes. These processes involve harvesting cells from the culture medium, lysing them to release intracellular PHA granules, and subsequently recovery and purifying the PHA [50]. These recovery processes are expensive and require high energy consumption and the use of nonenvironmentally friendly solvents, such as chloroform, and acetone [14,23]. The cost of the extraction processes ranges from 0.7 to 1.3 €/kg, which accounts for approximately 30% of the total PHA production costs (2.3–4.3 €/kg) [51,52]. To address this issue, recent studies have demonstrated that increasing cell size and altering cell morphology can lead to improved PHA accumulation and high cell density growth, which may lead to greater production and extraction efficiency and could significantly reduce recovery costs [53,54].

### 3.2. Customizing PHA Properties Is Challenging

The material properties of PHAs are largely influenced by the monomer composition, which can be altered by changing the type and composition of the monomers. Factors that affect monomer composition include randomness, molar mass, and dispersity [55]. PHA synthases determine the composition of PHA monomers and exist in four types: type I, II, III, and IV. Type I, III, and IV specifically polymerize scl-PHA, while type II preferentially polymerizes mcl/lcl-hydroxyacyl-CoA [56]. Currently, the diverse range of PHA structures is achieved by various PHA synthases with different specificities. The specificity of the PHA synthase leads to the formation of block copolymers in bacterial PHA synthesis. In addition, the monomer composition is influenced by the host bacteria, substrates, and fermentation conditions [57,58].

Different arrangements of scl-PHA and mcl-PHA in the polymer can form homopolymers, copolymers, and block copolymers with uniform monomer structures. The most commonly produced PHAs using wild-type bacteria are the homopolymer of *(R)*-3-hydroxybutyrate (3HB). However, the majority of PHAs produced using wild-type bacteria are copolymers of at least two different monomers. Unfortunately, natural PHA copolymers are amorphous elastic or viscous polymers with no significant application [59]. To address this limitation, metabolic-engineered strains have been developed to synthesize novel mcl-PHA homopolymers and block copolymers. However, this often requires specific carbon-chain-length fatty acid substrates [10,59].

As a result, customizing natural PHAs to meet specific requirements can be a highly challenging task, especially when incorporating monomers with distinct structures such as benzene rings, nitrogen, and sulfur-containing groups. In such cases, post-polymerization modifications are often required [9]. Therefore, the synthesis of new polyesters with specific properties and improved performance to meet the demands of industrial and medical applications remains an area that requires ongoing efforts.

### 3.3. The Complexity of Cellular Regulation

Wild-type strains of bacteria typically synthesize scl-PHA with much lower yields of mcl-PHA and lcl-PHA. However, PHA yield and composition are dynamically regulated by multiple genes. The low conversion of substrate to PHA is often due to the consumption of precursors by other pathways, leading to imbalances in PHA yield, composition, and molar mass [60]. To promote PHA production, excess carbon nutrients must be supplied. The regulation of PHA metabolism involves PHA granule-associated proteins (PGAPs), including PhaR, PhaM, PhaF, and PhaQ, which bind to related promoters of PHA biosynthetic genes to regulate their transcription to ensure PHA granule formation [61,62,63,64,65]. For example, PhaR and PhaM regulate PHB synthesis in *Cupriavidus necator* [66], while PhaF and PhaI, which encode the phasins, play important regulatory roles in *Pseudomonas putida* KT2440 [67]. In *Haloferax mediterranei*, the synthesis of PHBV (poly 3-hydroxybutyrate-co-3-hydroxyvalerate) is regulated by pps-like proteins, which repress PHA synthase (PhaC) expression [68]. Thus, the regulation of PHA synthesis can be modulated through modifications of regulatory proteins. While PHA synthesis has been demonstrated in a significant number of bacteria, there has been limited exploration and clear elucidation of the corresponding regulatory components. As a result, engineering applications in this field are still in their early stages. Despite extensive research into well-studied strains such as *Halomonas bluephagenesis* [69], *Pseudomonas putida* [67], and *Cupriavidus necator*, a substantial amount of metabolic regulatory information related to PHA synthesis remains elusive. Although metabolic-engineered strains have been studied to address this knowledge gap, it is even more challenging in the context of a complex in vivo system [70].

## 4. In Vitro PHA Synthesis

### 4.1. Exploration of PHA Synthetase

Cell-free synthetic biology, in conjunction with the prototyping design, may offer a promising approach for PHA synthesis. In the 1970s, research into the in vitro synthesis of PHA using purified enzymes [71] was carried out, primarily aimed at exploring the activity of PhaC to optimize the PHA synthesis capability of the strain. Since then, this approach has been widely adopted by researchers. Qi*,* et al. [72] purified the type II PHA synthases PhaC1 and PhaC2 from *Pseudomonas aeruginosa* and determined their kinetic parameters for PHA synthesis. A molar mass of 9.8 × 10^6^ g/mol of PHB was obtained when using *(R*,*S)*-3-hydroxybutyryl-CoA as a substrate. Additionally, PhaC activity can be inhibited by large amounts of CoA. The reaction pathway catalyzed by PhaC from acyl-CoA to PHA is a single step and provides direct and clear guidance for subsequent work with the in vitro synthesis of PHA.

Subsequently, other researchers developed cell-free synthesis methods that improved the polymer yield. Jossek and Steinbüchel [73] established an in vitro PHB three-enzyme biosynthetic system. In this system, the release of CoA was coupled with acetate, which was reused as an acetyl group donor catalyzed by acetyl-CoA synthase to generate 3-hydroxybutyrate. This method made the in vitro synthesis of PHB independent of the costly consumption of CoA. In subsequent research, Han*,* et al. [74] discovered a method for synthesizing non-natural PHA using a chemical-enzyme approach. Their system consisted of an organic phase of hexane and a buffered aqueous phase. In the aqueous phase, propionyl-CoA transferase, with pan-substrate activity, catalyzed the CoA transfer from acetyl-CoA to form a CoA-activated precursor for a PHA monomer with the release of acetate. During polymerization, CoA was released into the aqueous reaction phase and reacetylated in the intermediate phase. Ultimately, the maximum titer of PHB achieved was 1.2 g/L, which is comparable to that of natural producers [75]. Unconventional PHA cell-free synthesis schemes have also been reported, such as the use of class II and III PHA synthases to catalyze PHA surface coatings on hydrophobic carriers [76]. Additional reports related to cell-free PHA synthesis are summarized in Table 1.

### 4.2. In Vitro Multienzyme Systems for PHA Synthesis

Flexible and controllable multienzyme cascade systems have been employed to test entire metabolic pathways [84,85,86,87]. It is reassuring that the rapid growth of synthetic biology has largely contributed to the development of controllable cell-free synthesis systems, the ability to guide substrates toward desired products, and the effective combination of facilities that can freely assemble heterologous enzymes [88]. In many ways, the cell-free synthesis method is a promising strategy for PHA production.

In vitro multienzyme systems for PHA synthesis have been investigated by several research groups. In 1998, Jossek and Steinbüchel [73] established an extracellular PHB tri-enzyme system with acetate and 3HB as substrates, which could be prepared on a semi-preparative scale. Five years later, Satoh, Tajima, Tannai and Munekata [83] developed an improved new enzyme-catalyzed PHB synthesis system using five enzymes including β-ketothiolase (PhaA), NADPH-dependent acetoacetyl-CoA reductase (PhaB), PhaC, acetyl-CoA synthetase (ACS), and glucose dehydrogenase (GDH). In this system, acetyl-CoA was synthesized from acetate, CoA, and ATP through ACS, and then, two acetyl-CoA molecules were condensed by PhaA to synthesize acetyl-CoA, which was transformed into *(R)*-3-hydroxybutanyl-CoA (3HB-CoA) by PhaB. Finally, 3HB-CoA was polymerized and transformed into PHB by PhaC, with a titer of 1.12 mg/mL. Subsequently, cell-free PHA (mainly PHB) synthesis systems based on PhaA, PhaB, and PhaC were combined into multiple enzyme reaction modules. For instance, Opgenorth*,* et al. [89] designed a PBG (pentose–bifido–glycolysis) cycle that effectively converted glucose into acetyl-CoA and demonstrated its utility in PHB production. In a recent study, Li*,* et al. [90] developed an in vitro system that coupled plant cystoid membranes and five enzymes to synthesize PHB and regenerate NADPH. Furthermore, Zhang*,* et al. [91] demonstrated PHB production from methanol by utilizing the artificial synthetic acetyl-CoA (SACA) pathway. It is noteworthy that although the previous studies highlighted the usefulness of the acetyl-CoA synthesis, they did not focus on enhancing PHA yield. Nonetheless, the considerable yield and rate data collected in these studies pave the way for the future of in vitro PHA synthesis.

## 5. Advantages and Prospects for In Vitro PHA Synthesis

### 5.1. Advantages of In Vitro PHA Synthesis

Cell-free methods have enormous potential in accelerating the design–build–test (DBT) cycle by avoiding resource conflicts between cell growth and product synthesis [92]. Cell-free synthetic technology is a promising approach that can complement in vivo metabolic engineering [93]. For instance, by mixing the substrates and enzymes, the efficiency of the biosynthesis pathway can be conveniently optimized in vitro [70,94]. Moreover, cell-free catalysis offers significant advantages over the bottlenecks encountered in PHA synthesis, as outlined below:

#### 5.1.1. The Limitation of Cell Volume Is Eliminated in Cell-Free Catalysis

Intracellular crowding can be exacerbated by the accumulation of PHA particles, leading to the termination of PHA production [95]. Despite attempts to alter cell morphology [54,96], the constraint of cell volume remains a limitation. On the other hand, a cell-free system offers an advantage by allowing for the easy separation of PHA from the system. This eliminates the spatial constraint and enables the system to maintain its continuous productivity as long as possible [89].

#### 5.1.2. Real-Time Monitoring of Product Concentration Can Be Achieved

The real-time detection of intracellular PHA content remains a significant challenge, often requiring post-fermentation analysis due to the lack of effective methods. Despite attempts to develop biosensors for this purpose [97,98], technical challenges, such as response specificity and accuracy in complex cellular systems, continue to pose significant obstacles. In contrast, Opgenorth, Korman and Bowie [89] successfully achieved real-time monitoring of PHB content in an in vitro system by measuring the absorbance value at 600 nm, which guided glucose replenishment and resulted in a high conversion rate of 93.6% [89]. Presently, achieving yields or conversions close to the theoretical maximum is already common in in vitro synthetic systems [88,99,100].

#### 5.1.3. Significant Reduction in Product Separation Costs Can Be Achieved

The production cost of PHA is significantly impacted by the energy consumption required for cell crushing and the cost of solvents used for extraction [101,102]. This energy-intensive and environmentally unfriendly production process poses a major challenge for PHA production. In contrast, in vitro PHA synthesis, with simple composition and minimal interference from metabolites, offers a promising alternative. This method only requires centrifugation to obtain PHA products with high purity and low cost [103]. Therefore, in vitro PHA synthesis holds immense potential for the development of sustainable and cost-effective PHA production processes.

#### 5.1.4. Compatible with New Pathways and Heterologous Enzymes

Introducing new pathways and heterologous enzymes into cells is a technically challenging process due to the complex regulatory system inherent to cells. Compatibility issues often arise, resulting in suboptimal results for artificial pathways with high theoretical yields. Conversely, in vitro systems offer a highly compatible and straightforward approach to pathway development, making them a preferred choice for many researchers. Moreover, PHA, being a classical acetyl-CoA derivative, is frequently chosen as a product for new pathway development [91,104]. Therefore, in vitro PHA synthesis presents a promising avenue for the development of novel and efficient biopolymer production processes with improved compatibility and technical feasibility.

#### 5.1.5. The Monomer Composition Is Better Controlled

The customization of the monomer composition of intracellular PHAs presents a significant challenge that hinders the study of PHA material properties. However, in vitro PHA synthesis provides a promising solution to this challenge, allowing for better control over the polymerization process and monomer composition by combining specific substrates, enzymes, and polymerases in a cell-free catalytic system. Therefore, in vitro PHA synthesis offers a unique opportunity for the production of tailored biopolymers with specific and customizable properties, facilitating the study of PHA material properties and expanding their potential applications.

### 5.2. Challenges with In Vitro PHA Synthesis

Despite the advantages of cell-free methods in overcoming cell growth constraints [105], many challenges remain to be solved before PHA products can be commercially available on a large scale.

#### 5.2.1. Insufficient Diversity in Monomer Composition

Scl-PHA is primarily synthesized from acetyl-CoA through the PhaCAB pathway, whereas mcl/lcl-PHA synthesis typically involves either the fatty acid β-oxidation or fatty acid synthesis pathway (Figure 1). However, the implementation of the fatty acid synthesis and degradation pathway in vitro is a challenging task due to the requirement for complex coenzymes or acyl-carrying proteins. Although there have been numerous reports on the in vitro synthesis of PHB using acetyl-CoA as the sole precursor [89], progress in the in vitro synthesis of mcl-PHA has been limited. Additionally, the inhibitory effect of CoA on PhaC [72] poses another challenge that needs to be addressed in the in vitro synthesis of PHA.

#### 5.2.2. Insufficient Enzyme Activity and Stability

**Figure 2:**
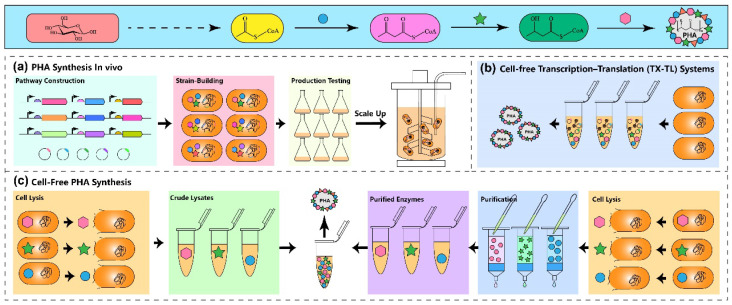
The patterns of PHA production in vivo and in vitro. (**a**) Traditional in vivo PHA production involves strain construction, metabolic pathways engineering, fermentation optimization, and process scaling up. (**b**) PHA synthesis using a transcription–translation system. In this system, the cell crude extract contains plasmid DNA and the enzymes required for PHA synthesis are first transcribed and translated in the cell crude extract to form the PHA synthesis pathway, which can synthesize PHA when the substrate is available. (**c**) Traditional cell-free PHA synthesis. The cell crude extracts were directly used as the reaction system (**left**), or mixing purified enzymes to form a cell-free system for PHA synthesis (**right**). In the figure, the blue circle indicates PhaA, the green star indicates PhaB, and the pink hexagon indicates PhaC.

Numerous in vitro multistep pathways have been developed to enhance product yield from low-cost substrates, using either crude extract or purified enzymes (Figure 2). However, the apparent activity and stability of enzymes in cell-free systems are often lower than those in live cells. To tackle this challenge, various potential solutions are worth exploring. For instance, employing thermostable enzymes [106] or improving enzyme stability through protein engineering [107,108], or multienzyme immobilization technology [109], could potentially address the issue of activity and stability. Furthermore, optimizing enzyme production efficiency and developing cost-effective production methods will play a crucial role in determining the commercial viability of in vitro PHA synthesis systems.

#### 5.2.3. Dependence on Energy and Cofactors

Cofactors such as ATP, NAD(P)^+^, CoA, and FAD^2+^ play an indispensable role in cell-free PHA biosynthesis [110]. However, in industrial biocatalysis, these cofactors can be expensive supplements. For instance, the cost of NADH is approximately $260 g^−1^ [111]. To enhance economic feasibility, researchers are striving to regenerate and reuse cofactors [112,113] or reduce the use of expensive ones [114,115,116,117]. Artificial analogs can substitute for expensive cofactors [118] and can be designed to be more stable than their natural counterparts while still being recognized by enzymes. The CoA cycle is crucial for PHA synthesis, but its dependence on chemicals for cofactor repair in vitro hinders CoA from being stable for extended periods [119]. More comprehensive reviews of cofactor and ATP regeneration systems have been reported [110,120,121].

### 5.3. Feasible Solutions (What to Work On)

The in vitro synthesis of PHA holds promising prospects, emphasizing the need to actively explore methods to tackle the challenges outlined above. Here, several feasible solutions that have the potential to reduce the cost of in vitro PHA synthesis have been compiled or designed. It is anticipated that these efforts will lead to the enhancement of cost-effectiveness in the entire process of in vitro PHA production and the facilitation of the mitigation of future market challenges.

#### 5.3.1. Utilization of Unconventional Substrates

Currently, the utilization of cell-free synthetic systems is limited due to the high cost of raw materials. To compete with petroleum-based products, it is essential to reduce the material cost. One promising approach is to introduce nonconventional substrates, such as CO_2_, CH_4_, and lignocellulose, into the cell-free synthetic system. This can be achieved by incorporating a degrading absorption module [122,123] that enables the utilization of nonconventional substrates. However, the introduction of these substrates may alter the composition of the PHA monomer, resulting in PHA products with varying properties. This can pose additional challenges and complexity, but it also presents an opportunity to expand monomer diversity and create new materials with unique properties.

#### 5.3.2. Low-Cost Acquisition and Stability Modification of Enzymes

The use of purified enzymes in cell-free biotechnology is a costly and time-consuming process. A viable solution to this issue is whole-cell catalysis, which involves the use of unpurified cell extracts and lower-quality enzymes, making it a more accessible and affordable option [88]. This approach involves expressing the required enzymes in the cells and then breaking the cell walls to release them into the buffer system. However, it is important to note that developing low-cost purification methods is a more promising strategy. To improve the stability of enzymes in the PHA in vitro synthesis pathways, several techniques such as directed evolution, enzyme immobilization, and screening of natural thermostable enzymes can be employed in conjunction with developing low-cost enzyme purification technology to achieve efficient PHA production. Immobilized cell-free systems immobilize enzymes and cofactors on or within a carrier material, resulting in a series of biochemical reactions with reduced or prevented enzyme migration [124]. Compared to free or soluble enzymes, immobilized enzymes possess higher stability, reusability, and simplified recovery and purification processes [22]. Moreover, by developing efficient protein expression and secretion systems [125], it is possible to circumvent the energy and materials consumption and enzyme activity damage associated with cell lysis and protein purification. The continuous optimization of cell surface display technology [126,127] and the immobilization of multiple cells [128] can facilitate the creation of multienzyme systems that reduce PHA production costs through improved activity, stability, and reusability of enzymes.

#### 5.3.3. Cofactor Regeneration

In cell-free systems, the use of cofactors can be costly. Consequently, many efforts have been made in developing strategies for balancing and regenerating cofactors. One approach is to integrate coenzyme reactions, while another is to utilize light and electrical energy for regeneration. Additionally, using inexpensive substrate cofactors to regenerate spent cofactors and designing cofactor-free pathway systems are also viable options. For example, the regeneration of NAD(P)^+^ can be accomplished through the use of NAD(P)H oxidase (Nox), which is formed by hydration and oxidizes NAD(P)H to NAD(P)^+^ [89]. NAD^+^ can be recovered from NADH and less expensive substrates, such as glucose, by using coupled reactions that involve glucose dehydrogenase [129]. Regenerating ATP can be achieved through the use of polyphosphate kinase (Ppk) [112], which accepts inexpensive polyphosphates and synthesize ATP from ADP or AMP in ATP-dependent reactions [123,130]. Clean energy sources such as light and electricity are recommended due to their cost-effectiveness. Li, Wei, Zhang, Liu, You and Zhu [90] utilized light by incorporating thylakoid membranes from spinach leaves into the system to provide ATP and NADP+ for the PHA synthesis pathway. Additionally, CoA can be released from the condensation and polymerization catalyzed by PhaA and PhaC in the PHB synthesis pathway and then reused in acetyl-CoA synthesis [83].

#### 5.3.4. Modeling In Silico Provides Quantitative Guidance

Computational modeling offers a quantitative framework for pathway validation and optimization by predicting intracellular metabolite concentrations and reaction fluxes [131]. In cell-free processes, changes in reaction rates and intermediate concentrations can be attributed to enzymatic activities. Kinetic pathway modeling can facilitate the adjustment of enzyme, substrate, and cofactor concentrations, contributing to our rational understanding of cell-free systems [132,133]. For instance, Rollin*,* et al. [134] fitted a nonlinear kinetic model to experimental data, achieving a complete conversion of glucose and xylose to H_2_ and CO_2_ in a cell-free system. Through the analysis of enzymes with the greatest control coefficient for the desired reaction flux, the optimized enzyme usage led to a production rate of 32 mmol H_2_·L^−1^·h^−1^. Horvath et al. developed a genome-scale cell-free protein synthesis (CFPS) kinetic model to predict protein production pathways [135].

Moreover, genome-scale constraint-based models, such as stoichiometric metabolic network models, enzyme-constrained models [136], and thermodynamically constrained models [137,138], have been applied to guide pathway selection and to perform thermodynamic driving force and enzyme cost analyses for products and biomass synthesis. These models have broad applicability and have been applied to, or are expected to serve in, the in vitro PHA synthesis process [139]. Meanwhile, genome-scale kinetic modeling [140], or large-scale whole-cell models [141], have provided promising visions for rational product synthesis. The rapid development of artificial intelligence in parameter mining also provides greater opportunities for the deep integration of experimentation and simulation [142].

#### 5.3.5. Exploring the Modular PHA Synthesis

**Figure 3:**
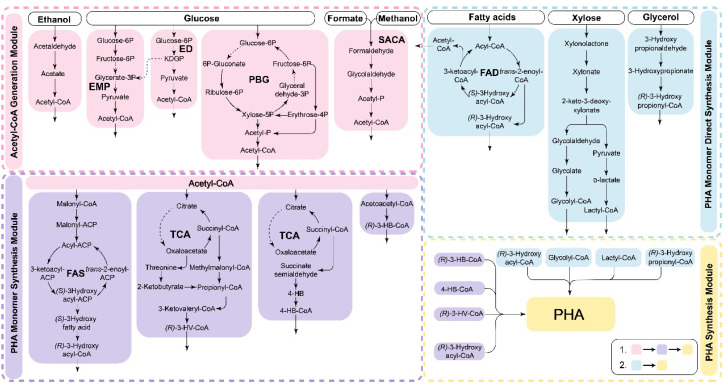
The in vitro modular assembly of synthetic PHA. The different metabolic modules are assembled and coupled to synthesize PHA using different substrates. Cell-free PHA synthesis can be achieved in vitro by assembling various metabolic modules and then coupling the PHA synthesis pathway to achieve the utilization of different substrates. The process typically involves generating acetyl-CoA modules and then combining them with the acetyl-CoA utilization pathway to synthesize the precursors of PHA. The enzymes related to central metabolism exhibit high activity, leading to a high rate of acetyl-CoA production and ensuring a sufficient substrate supply for the subsequent PHA synthesis pathway. While the utilization of fatty acids can directly lead to the synthesis of mcl-PHA, non-fatty acid substrates usually need to be converted into acetyl-CoA before participating in the synthesis of PHA. PBG: pentose, bifido, and glycolysis pathway [89]; SACA: synthetic acetyl-CoA pathway [104].

The synthesis pathway of PHA exhibits significant flexibility and modularity (as shown in Figure 3). Modular PHA synthesis can be achieved by utilizing the modular assembly of multienzyme systems through cell-free metabolic pathways to construct complete PHA synthesis pathways. Various substrates are metabolized through pathways such as central carbon metabolism, β-oxidation, and fatty acid synthesis (FAS) to provide precursors for PHA production [9]. Scl-PHA synthesis begins with acetyl-CoA as the precursor, which is polymerized by PhaA, PhaB, and PhaC. For mcl- and lcl-PHA synthesis, various intermediate products of the fatty acid degradation and synthesis pathways can also serve as precursors. When fatty acid substrates undergo β-oxidation degradation, reducing the activity of FadB and FadA can redirect intermediate metabolites toward mcl-PHA synthesis [9]. Non-fatty acid carbon sources, such as glucose, xylose, glycerol, ethanol, and one-carbon metabolites, require a connection between the substrate assimilation pathways and PHA monomer precursor synthesis pathways, such as acetyl-, glycolyl-, lactyl-, and various hydroxylated fatty acyl-CoAs [143,144]. The modular assembly approach is particularly advantageous because it can efficiently incorporate new reactions and pathway modules, resulting in the rapid formation of novel synthesis routes. This approach holds great promise for the development of new production modes that can surpass original yield limitations, ultimately leading to reduced costs associated with PHA production.

## 6. Prospect

The field of PHA production has made significant advancements and breakthroughs, with many studies and engineering efforts aimed at improving PHA synthesis efficiency. Despite the complexity of metabolic flow still posing a challenge for this progress, cell-free technology offers high controllability and operability, making it a promising approach for PHA synthesis. The cell-free prototype strategy complements in vivo workflow by enabling the determination and optimization of PHA production strategies. The exploration of new pathways for cell-free PHA synthesis represents a novel approach to PHA production. While there are still many challenges to be addressed in PHA synthesis in vitro, the ongoing evolution and optimization of solutions offer promising prospects for the future of cell-free PHA synthesis.

## Figures and Tables

**Table 1:** Summary for PHA synthesis exploration in vitro.

Substrates	Enzymes	Source of Enzymes	Products	Titer (mg/mL)	Conversion Rate (%)	Average M (g/mol)	Ref.
*(R*,*S)*-3HD-CoA	PhaC1, PhaC2	*P. aeruginosa*	P(3HD)	0.068	12	9.8 × 10^6^	[72]
*(R)*-LA and *(R)*-3HB, acetyl-CoA	PhaC1_SG_, PhaC2_SG_ LPE, CoA-transferase, and acetyl-CoA-synthetase	*Pseudomonas* sp. SG4502 and *Pseudomonas* sp. 61-3	P(LA-co-3HB)	0.77	/	2.7 × 10^5^	[77]
D-(-)-3-hydroxybutyric acid	acetyl-CoA-synthetase, PCT, and PhaEC_cv_	*C. vinosum and C. propionicum*	PHB	2.75	73	1–2 × 10^6^	[73]
*(R)*-3-Hydroxyoctanoyl-CoA	PhaC1_PP_ and PhaEC_Av_	*P. putida and Allochromatium vinosum*	PHB	/	/	/	[76]
HB−NAC and HB−CoA	PhaEC_Av_	*A. vinosum*	PHB	/	/	5.84–7.97 × 10^4^	[78]
β-butyrolactone	FadB1x, PhaC_Da_, and PhaJ_Ac_	*A. caviae, D. acidovorans, and P. putida* KT2440	PHB	1.1575	27	1.2 × 10^5^	[79]
thiophenyl *(R)*-3-hydroxybutyrate	PhaC	*R. eutropha*	PHB	0.36	47	1.6 × 10^6^	[80]
*(R)*-2HB, AcETG, *(R)*-3HB	PHA synthase I, PHA synthase II, and PHA synthase IV PCT (propionate CoA transferase)	*R. eutropha, Pseudomonas* sp. SG4502, *Synechocystis* sp. PCC6803, *Bacillus* sp. INT005, and *C. propionicum* JCM1430	P(2HB), PHB	1.2	/	1.00 × 10^5^	[74,75]
Glucose, NADPH, acetoacetyl-CoA	PHA synthase, acetoacetyl-CoA reductase, and glucose dehydrogenase	*A. eutrophus*	PHB	/	/	10 × 10^6^	[81]
crotonic anhydride	PhaJ and PhaC	*A. caviae* FA440 *and R. eutropha* H16	PHB	/	58	6.4 × 10^6^	[82]
Acetate, CoA	ACS, PhaA, PhaB, and PhaC	*E. coli* JM109 *and R. eutropha* H I6	PHB	1.12	45.5	6.64 × 10^6^	[83]

Note: M indicates molar mass.

## Data Availability

Not applicable.
